# Protective effect of zinc against A2E-induced toxicity in ARPE-19 cells: Possible involvement of lysosomal acidification

**DOI:** 10.1016/j.heliyon.2024.e39100

**Published:** 2024-10-11

**Authors:** Jeong A. Choi, Bo-Ra Seo, Jae-Young Koh, Young Hee Yoon

**Affiliations:** a Neural Injury Research Center, Asan Institute for Life Sciences, Asan Medical Center, University of Ulsan College of Medicine, Seoul, South Korea; b Department of Neurology, Asan Medical Center, University of Ulsan College of Medicine, Seoul, South Korea; c Department of Ophthalmology, Asan Medical Center, University of Ulsan College of Medicine, Seoul, South Korea

**Keywords:** Lysosome dysfunction, Zinc, Retinal pigment epithelium, Age-related macular degeneration

## Abstract

A key pathogenic mechanism of dry age-related macular degeneration (AMD) is lysosomal dysfunction in retinal pigment epithelium (RPE) cells, which results in the accumulation of lipofuscins such as A2E (N-retinylidene-N-retinylethanolamine) that further compromises lysosomal function. This vicious cycle leads to cell death and poor visual acuity. Here, we established an *in vitro* model of AMD by treating a human RPE cell line (ARPE-19) with A2E and examined whether raising zinc levels confers protective effects against lysosomal dysfunction and cytotoxicity. MTT assay showed that A2E induced apoptosis in ARPE-19 cells. pHrodo™ Red fluorescence staining showed that lysosomal pH increased in A2E-treated ARPE-19 cells. Treatment with a zinc ionophore (clioquinol) reduced A2E accumulation, restored lysosomal pH to the acidic range, and reduced A2E-induced cell death, all of which were reversed by the addition of a zinc chelator (TPEN). Consistent with the *in vitro* results, subretinal injections of A2E in mouse eyes resulted in the death of RPE cells as well as lysosomal dysfunction, all of which were reversed by co-treatment with clioquinol. Our results suggest that restoring the levels of intracellular zinc, especially in lysosomes, would be helpful in mitigating A2E-induced cytotoxic changes including lysosomal dysfunction in RPE cells in the pathogenesis of AMD.

## 1 Introduction

Dysfunctions in the retinal pigment epithelium (RPE) and photoreceptors are the hallmarks of age-related macular degeneration (AMD), the most prevalent cause of permanent vision loss in individuals over the age of 60 [[1], [2], [3], [4]]. While AMD does not typically lead to complete blindness, the loss of central vision can significantly impair the ability to perform tasks that require fine visual detail. The progression of AMD varies among individuals, with some experiencing a gradual decline in vision, while others may notice a more rapid deterioration [5]. Based on the existence of neovascularization, age-related macular degeneration (AMD) is categorized into non-exudative (dry AMD) and exudative (wet AMD). Dry AMD, which accounts for nearly 90 % of all AMD cases [1,[6], [7], [8]], is characterized by the accumulation of insoluble extracellular aggregates called drusens between the RPE and the Bruch's membrane, which may compromise functions of photoreceptors and RPE cells and leads to decreased visual acuity [2,[9], [10], [11], [12]].

RPE cells are phagocytic cells that engulf photoreceptor outer segments (POS) and degrade them via lysosomal degradation to reuse proteins and lipids [2,11,13,14]. During the lysosomal degradation process of photoreceptor outer segments, RPE cells produce lipofuscin, which are aggregates of fluorescent compounds derived from vitamin A and oxidized proteins and lipids. One of the key components of drusen and lipofuscin is A2E (N-retinylidene-N-retinylethanolamine) [[15], [16], [17], [18], [19], [20]], which is derived from all-trans-retinal and phosphatidylethanolamine. As RPE cells age, lysosomal degradation becomes dysfunctional and lipofuscin accumulates [[21], [22], [23], [24]]. Importantly, a substantial body of evidence suggests that lipofuscin and its major fluorophore, A2E, increase lysosomal pH and further reduce the degradative capacity of the RPE [[24], [25], [26], [27], [28]], thus contributing to the pathogenesis of AMD. The lysosomal dysfunction induced by lipofuscin may lead to further accumulation of lipoprotein aggregates [22,[29], [30], [31], [32]], thereby establishing a vicious cycle in the pathogenesis of AMD. Consistent with these ideas, previous studies have demonstrated that the alkalinization of lysosomes plays a key pathogenic role in AMD and that lysosomal reacidification may be a viable therapeutic strategy for AMD [22,30,[33], [34], [35], [36]].

Zinc is an essential trace element involved in numerous biological processes, including protein synthesis and enzyme function [37,38]. In the retina, zinc plays a crucial role in maintaining the structure and function of photoreceptors and the retinal pigment epithelium (RPE) [39,40]. Recent studies have provided strong evidence that zinc enhances lysosomal function. Kim et al. demonstrated that zinc enhances autophagic flux and lysosomal function by activating transcription factor EB (TFEB) and promoting the assembly of V-ATPase, a critical enzyme for lysosomal acidification and function [41]. Additionally, we recently showed that clioquinol (ClioQ), a potent zinc ionophore, effectively reacidifies lysosomal pH and overcomes the autophagy arrest induced by chloroquine in ARPE-19 cells, a widely used human RPE cell line [42]. In this study, we investigated the effects of zinc on the acidity and degradative function of the lysosome in A2E-treated ARPE-19 cells. To modulate zinc levels, we performed experiments using the zinc ionophore clioquinol and the zinc-specific chelator TPEN [[43], [44], [45]]. Furthermore, we tested the effects of zinc on A2E clearance in mice injected subretinally with A2E. Here, we report that raising intracellular zinc levels with clioquinol resulted in a reduction of A2E accumulation within the RPE.

## 2 Materials and methods

### 2.1 Cell culture

ARPE-19 cells were obtained from the American Type Culture Collection (CRL-2302; Manassas, VA, USA). Dulbecco’s Modified Eagle Medium (DMEM) was used to culture the ARPE-19 cells in a humidified 37 °C incubator with 5 % CO_2_. DMEM was supplemented with 1 % penicillin-streptomycin (Lonza, Allendale, NJ, USA), 10 % fetal bovine serum (Invitrogen, Carlsbad, CA, USA), and Nutrient Mixture F-12 (Invitrogen) [42,46]. Cell passage maintenance and subculture were performed by referring to ATCC's ARPE-19 cell product sheet. All ARPE-19 cells were cultured on tissue culture-treated coated plates. ARPE-19 cells (3.0 × 10^5^ cells/well) were seeded into 6-well cell culture plates (Thermofisher, Waltham,MA, USA) until 80 % confluence, after which they were used for experiments.

### 2.2 Chemicals

N,N,N’,N’-Tetrakis(2-pyridylmethyl)ethylenediamine (TPEN), ZnCl_2_, and clioquinol (ClioQ) were purchased from Sigma (St. Louis, MO, USA). N-retinylidene-N-retinylethanolamine (A2E) was purchased from Abta Bio (Seoul, Korea). 3-(4,5-Dimethylthiazol-2-yl)-2,5-diphenyltetrazolium bromide (MTT) was purchased from Amresco (Wayne, PA, USA).

### 2.3 Live-cell confocal microscopy

For live-cell imaging, ARPE-19 cells were cultured on a cover glass 24 h before the experiment [46]. Then, the cells were transferred to a live imaging solution or MEM and observed under a confocal microscope at an excitation wavelength of 488 nm and emission wavelength ranging from 490 to 575 nm. LSM780 Confocal Live-Cell Imaging System (Carl Zeiss, Oberkochen, Germany) was utilized for obtaining live-cell confocal images. All fluorescence intensities are expressed in arbitrary units.

### 2.4 Lysosomal pH assessment

The ARPE-19 cells were cultured in glass-bottom plates until they reached 60 % confluence. In a humidified 5 % CO_2_ incubator (37 °C), the cells were stained using pHrodo™ Red AM intracellular pH indicator (Invitrogen, P35372) for 30 min; then, the cells were moved to a confocal live-cell chamber. The experimental conditions were as follows: A2E alone, A2E plus 1 μM ClioQ and 0.5 μM Zn (ZnClioQ), or A2E plus ZnClioQ and 0.5 μM TPEN. pHrodo™ Red AM was observed at an optimal excitation/emission spectra of 560/585 nm. LSM780 Confocal Live-Cell Imaging System (Carl Zeiss) was used to obtain confocal images.

### 2.5 Immunocytochemistry

After fixing the cells in 4 % paraformaldehyde, we washed the cells using PBS. Then, the cells were incubated with a permeabilizing and blocking PBS solution containing 1 % bovine serum albumin and 0.2 % Triton X-100 for 1 h. Immunocytochemistry was performed following the protocols published in a previous study [42,46,47], with some modifications. The cells were first incubated with the antibody against LAMP-1 (Abcam, ab24170, 1:250) for 24 h at 4 °C and then with a secondary antibody (Invitrogen, A31572, 1:500) prior to confocal visualization (LSM780; Carl-Zeiss). For visualizing PM (plasma membrane), we used the Alexa Flour 594 conjugate wheat germ agglutinin (WGA) dye (Cat. W11262, Invitrogen^TM^).

### 2.6 Cathepsin B activity assay

ARPE-19 cells were treated with A2E alone, A2E plus ZnClioQ, or A2E plus ZnClioQ and TPEN for 1 h or 6 h. In a humidified 37 °C 5 % CO_2_ incubator, the cells were stained with Magic Red Substrate using the Magic Red® Cathepsin B Assay Kit (Immunochemistry, Bloomington, MN, USA) for 15 min; subsequently, the cells were transferred to a confocal live-cell chamber. Cathepsin B activity was observed at an optimal excitation wavelength of 592 nm and an emission wavelength of 628 nm.

### 2.7 Intracellular A2E concentration quantification

To measure the amount of intracellular A2E, ARPE-19 cells were treated with A2E alone, A2E plus ZnClioQ, or A2E plus ZnClioQ and TPEN for 1 h. Then, A2E-containing media was suctioned, and cells were thoroughly washed out with PBS. Experimented cells were harvested with 80 % methanol, and placed in an internal standard solution for LCMSMS. After the centrifuge, the supernatant was collected, and chloroform was added. After one more centrifuge, the separated aqueous layer is collected. A2E concentrations were analyzed using Ultimate3000 (LC, Dionex) and Orbitrap XL (MS, Thermo Fischer Scientific) equipment in the Metabolomics core at the Convergence Medicine Research Center, Asan Medical Center.

### 2.8 Cell death assessment

To test the toxicity of A2E, the cells were treated with A2E alone, A2E plus, ZnClioQ, or A2E plus ZnClioQ and TPEN for 24 h A2E-induced cell death was quantified by MTT assay, which measures the metabolic activity of cells through colorimetry. In summary, the medium from each well was eliminated, and 300 μl of MTT solution (0.5 mg/ml) was added to each well. The wells were then incubated at 37 °C for 1 h. The MTT solution was then aspirated, and 200 μl of dimethyl sulfoxide (DMSO) was added to each well to dissolve the reduced formazan crystals produced by metabolically active cells. An automated microplate reader (UVmax; Molecular Devices, San Francisco, CA, USA) was used to measure the absorbance at 590 nm. MTT assay was performed following the protocols published in a previous study [48,49], with some modifications.

### 2.9 Animals

The animal experiment protocol was approved (approval number: 2021-12-280) by the Institutional Animal Care and Use Committee (IACUC) of Asan Institute for Life Sciences, University of Ulsan College of Medicine (Seoul, Korea). All procedures were conducted in accordance with the Asan Institute for Life Sciences, University of Ulsan College of Medicine IACUC guidelines, and the ARRIVE guidelines. Male C57BL/6J mice weighing 22–25 g (8 weeks old) were purchased from JA Bio (Seoul, Korea). The mice were reared under a 12 h light/dark cycle and a temperature of 24 °C ± 0.5 °C. We performed subretinal injections with A2E (100 μM/3 μl/eye) or A2E plus ZnClioQ (1 μM ClioQ and 0.5 μM Zn/3 μl/eye) and saline alone (vehicle). Twenty-four hours after the injection, mice were sacrificed and eyeballs were obtained. Subretinal injection was performed following the protocols published in a previous study [48].

### 2.10 Immunohistochemistry

Using a cryostat, retinal sections were produced and placed onto glass slides. We used 4 % paraformaldehyde to fix the slides before rinsing them in PBS. Then, a PBS solution containing 1 % bovine serum albumin and 0.2 % Triton X-100 was used to permeabilize and block the slides [47,50]. After a 24-h incubation at 4 °C with an anti-LAMP1 antibody (Abcam, 1:250), Alexa 555 Fluor-conjugated secondary antibody (Invitrogen, 1:500) was used for further incubation. The resulting slides were visualized using confocal microscopy (LSM780; Carl-Zeiss). T-PMT (transmitted-PMT) images were obtained through a confocal microscopy system along with fluorescence images.

### 2.11 Tissues preparation for HPLC

The anterior segment of the eye, vitreous, and retina were carefully removed and discarded, and only RPE/choroid tissue was collected. Tissue samples were stored at −80 °C until use. Then, the amount of A2E was detected in the tissue and analyzed with the same equipment as in the cell.

### 2.12 Statistical analysis

Statistical analyses were performed similarly to a previous study [47,50]. All results had a normal distribution and are presented as mean ± standard error of the mean (SEM). Student's t-test was used to evaluate the significance of differences between the two groups. A one-way ANOVA test was used to evaluate the significance of differences between more than three groups. One-way ANOVA followed by post hoc Tukey’s multiple comparison test was performed using Graph Pad Prism. Statistical significance was set at *∗P < 0.05, ∗∗P < 0.01,* ^*#*^*P <0.005*, and ^*##*^*P < 0.001*. All statistical analyses were performed using GraphPad Prism 8 (GraphPad Software Inc., CA, USA) and SigmaPlot software version 10.0 (Systat Software, Inc., San Jose, CA, USA).

## 3 Results

### 3.1 *In vitro* model of AMD

**Fig. 1:**
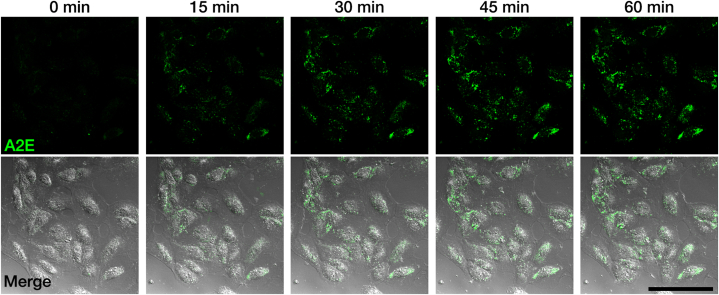
Establishment of an *in vitro* model of age-related macular degeneration. Confocal live images showing the accumulation of A2E (green) in ARPE-19 cells following treatment with 100 μM A2E. Original magnification, × 400; scale bar, 100 μm.

Considering that the accumulation of lipofuscin and A2E is a key factor in the pathogenesis of AMD, we developed an *in vitro* model of AMD by treating ARPE-19 cells with A2E. We treated ARPE-19 cells with 100 μM A2E. Considering that A2E exhibits autofluorescence [[51], [52], [53]], we tracked the accumulation of A2E in ARPE-19 cells with live-cell confocal microscopy (Fig. 1 and Supplementary Fig. S1). We observed that A2E, initially present in the media, gradually accumulated as aggregates in ARPE-19 cells over the course of an hour.

### 3.2 A2E accumulation reduces lysosomal acidity

**Fig. 2:**
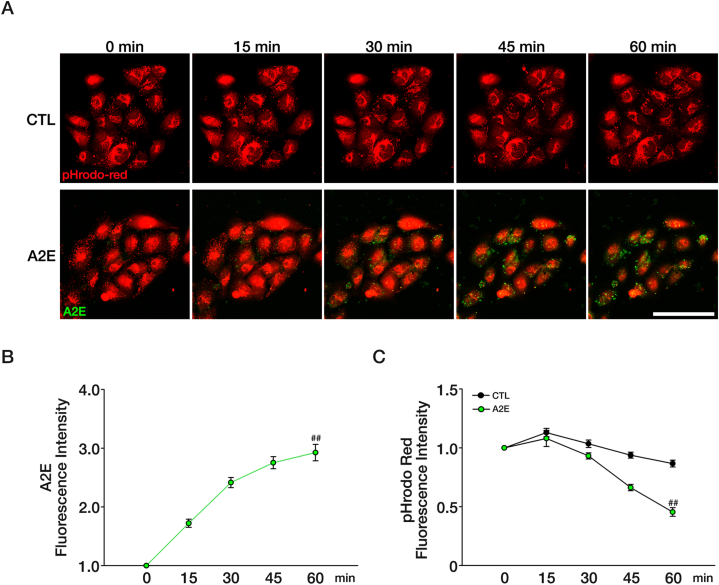
A2E accumulation reduces lysosomal acidity. (A) Time-wise pH changes in pHrodo™ Red AM-stained ARPE-19 cells according to treatment with 100 μM A2E. Accumulation of A2E is shown in green dots. Original magnification, × 400; scale bar, 100 μm. (B) Quantification of A2E fluorescence using ROI analysis (n = 7, ^##^*P* < 0.001 between 0 min and 60 min, Student's t-test). (C) Quantification of pHrodo™ Red AM fluorescence using ROI analysis (CTL; n = 13, A2E; n = 7, ^##^*P* < 0.001 comapred to CTL and A2E group at 60 min, Student's t-test).

Because lysosomal degradation is often compromised in AMD, we investigated the possibility that A2E may directly induce lysosomal dysfunction in ARPE-19 cells. We first examined the changes in lysosomal pH by treating pHrodo™ Red AM, a pH-sensitive lysosome-specific fluorescent dye, to ARPE-19 cells. In cells treated with A2E, we observed a gradual increase in A2E fluorescence (Fig. 2A and B) and a concurrent decrease in pHrodo™ Red fluorescence, which were not evident in untreated control cells (Fig. 2A–C). These findings suggest that A2E accumulation, likely in the lysosomes of ARPE-19 cells, is closely associated with the alkalinization of lysosomes.

### 3.3 Zinc treatment reacidifies lysosomes in ARPE-19 cells

Several studies demonstrated that zinc supplement is an effective measure to ameliorate AMD [[54], [55], [56], [57]]; yet, the mechanism underlying the zinc-mediated protection against AMD has not been elucidated. Previously, we demonstrated that zinc ionophores can reacidify lysosomes in diverse pathological conditions by increasing intracellular zinc levels, especially in lysosomes [30,58]. We thus speculated that increasing zinc levels may help reduce the accumulation of AMD-associated toxic materials such as A2E.

**Fig. 3:**
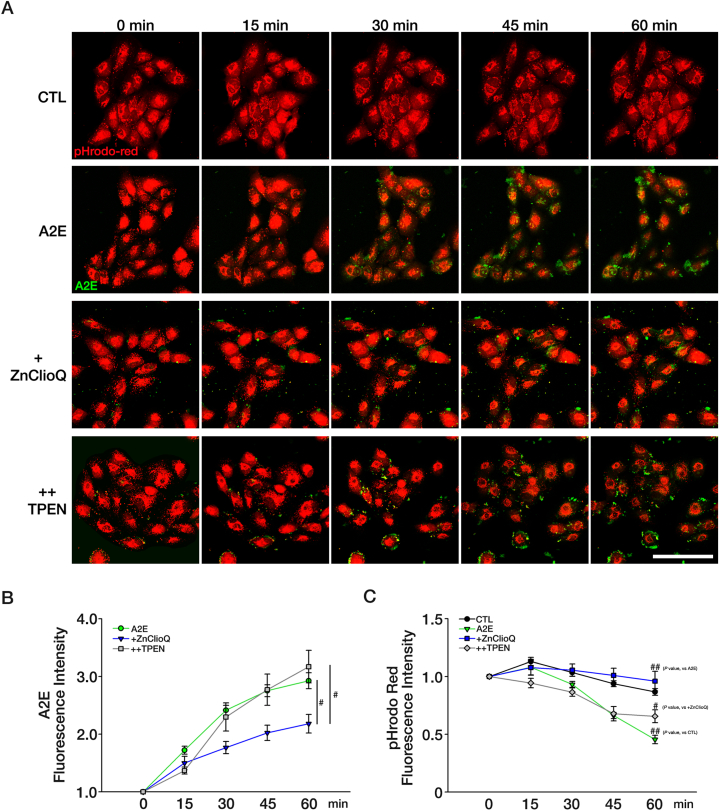
Zinc treatment reacidifies lysosomes in ARPE-19 cells. (A) Time-wise pH changes in pHrodo™ Red AM-ARPE cells treated with 100 μM A2E alone, A2E plus 1 μM ClioQ and 0.5 μM Zn (ZnClioQ), or A2E plus ZnClioQ and 0.5 μM TPEN. Original magnification, × 400; scale bar, 100 μm. (B) Quantification of A2E fluorescence using ROI analysis (A2E; n = 7, +ZnClioQ; n = 13, ++TPEN; n = 9, ^#^*P* < 0.005, one-way ANOVA test). (C) Quantification of pHrodo™ Red AM fluorescence using ROI analysis (CTL; n = 13, A2E; n = 7, +ZnClioQ; n = 13, ++TPEN; n = 9, ^#^*P* < 0.005, ^##^*P* < 0.001, one-way ANOVA test). ∗Experimental Group = CTL; A2E; +ZnClioQ (A2E + ZnClioQ); ++TPEN (A2E + ZnClioQ + TPEN).

To examine the effect of zinc on A2E-induced changes in lysosomal pH, we performed live-cell imaging using the zinc ionophore clioquinol (ClioQ) (Fig. 3, Supplementary Fig. S2). Treatment of A2E-exposed ARPE-19 cells with 1 μM ClioQ plus 0.5 μM Zn (ZnClioQ) markedly increased the intensity of pHrodo™ Red AM fluorescence (Fig. 3A–C). This result indicates that the pH of the lysosomal was brought back to the acidic range. Notably, the effect of ZnClioQ on lysosomal pH entirely eliminated zinc chelation with TPEN treatment, indicating that the acidification was mediated by intracellular zinc (Fig. 3A–C). At the same time, ZnClioQ treatment decreased the accumulation of A2E in the ARPE-19 cells and the addition of TPEN abrogated the effect of ZnClioQ on the reduction of A2E accumulation (Fig. 3A and B). These results suggest that reacidification of lysosomes and normalization of their degradative function by zinc may help degrade A2E and reduce its accumulation.

### 3.4 Zinc facilitates the degradation of A2E

**Fig. 4:**
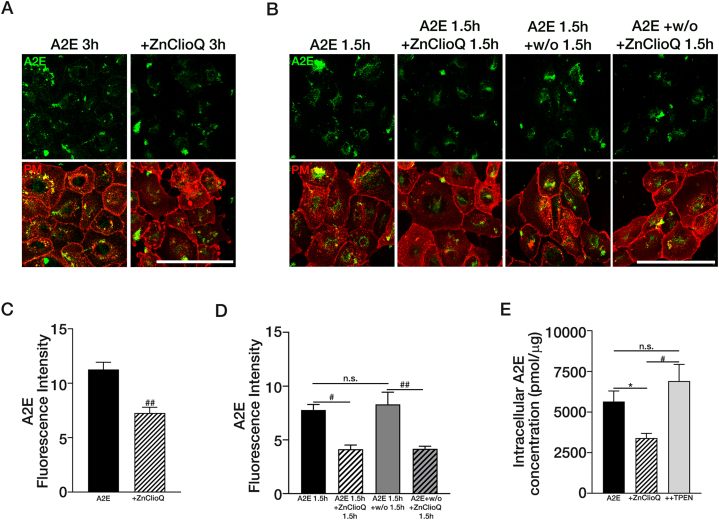
Zinc facilitates the degradation of A2E. (A) Confocal images of ARPE-19 cells that were treated with A2E or treated A2E with ZnClioQ for 3 h (Red; PM = plasma membrane). Original magnification, × 630; scale bar, 100 μm. (B) Confocal images of ARPE-19 cells. To investigate whether degradation of A2E is facilitated by zinc in intracellular A2E accumulation, the experimental group was treated with A2E for 1.5 h, then with ZnClioQ for 1.5 h, washed out (w/o), and then either left untreated or treated with ZnClioQ for 1.5 h. The plasma membrane is shown in red. Original magnification, × 630; scale bar, 100 μm. (C) Quantification of A2E fluorescence (A) using ROI analysis (CTL; n = 7, A2E; n = 7, +ZnClioQ; n = 7, ^##^*P* < 0.001, one-way ANOVA test). (D) Quantification of A2E fluorescence (B) using ROI analysis (A2E; n = 9, +ZnClioQ; n = 10, A2E + w/o; n = 10, w/o + ZnClioQ; n = 13, ^#^*P* < 0.005, ^##^*P* < 0.001, one-way ANOVA test). (E) Quantification of intracellular amount of A2E using LCMSMS (A2E; n = 5, +ZnClioQ; n = 4, ++TPEN; n = 4, ∗*P* < 0.05, ^#^*P* < 0.005, one-way ANOVA test). ∗Experimental group = A2E; + ZnClioQ (A2E + ZnClioQ); ++TPEN (A2E + ZnClioQ + TPEN).

As described above, we observed a decrease in internalized A2E by ZnClioQ in ARPE-19 cells. Compared to A2E-treated ARPE-19 cells (control), those co-treated with ZnClioQ showed a reduction in A2E accumulation during the course of 3 h (Fig. 4A–C). To determine whether the reduction of intracellular A2E levels resulted from decreased endocytosis or increased degradation, we allowed ARPR-19 cells to take up A2E for 90 min and then washed out extracellular A2E (Fig. 4B–D), thus ensuring that the initial intracellular A2E levels were the same. Beginning with the same amount of endocytosed A2E, we either sham-washed the ARPE-19 cells or treated them with ZnClioQ. Compared with control cells, ZnClioQ-treated ARPE-19 cells showed substantially reduced A2E fluorescence (Fig. 4D), which strongly supports the possibility that ZnClioQ reduces intracellular A2E by promoting lysosomal degradation rather than reducing its uptake.

To directly quantify the accumulation of A2E in intracellular space, we analyzed the cell lysates using the LCMSMS method (Fig. 4e, Supplementary Fig. S3). One hour after A2E treatment of ARPE-19 cells, cellular levels of A2E reached as high as 5 nmol/μg protein in cell lysates. Consistent with the above fluorescence measurements data (Fig. 4C and D), ZnClioQ treatment substantially reduced intracellular accumulation of A2E by approximately 40 % (Fig. 4E). Again, TPEN completely blocked the effect of ZnClioQ.

### 3.5 Zinc-induced changes in lysosome enzymatic activity in ARPE-19 cells

**Fig. 5:**
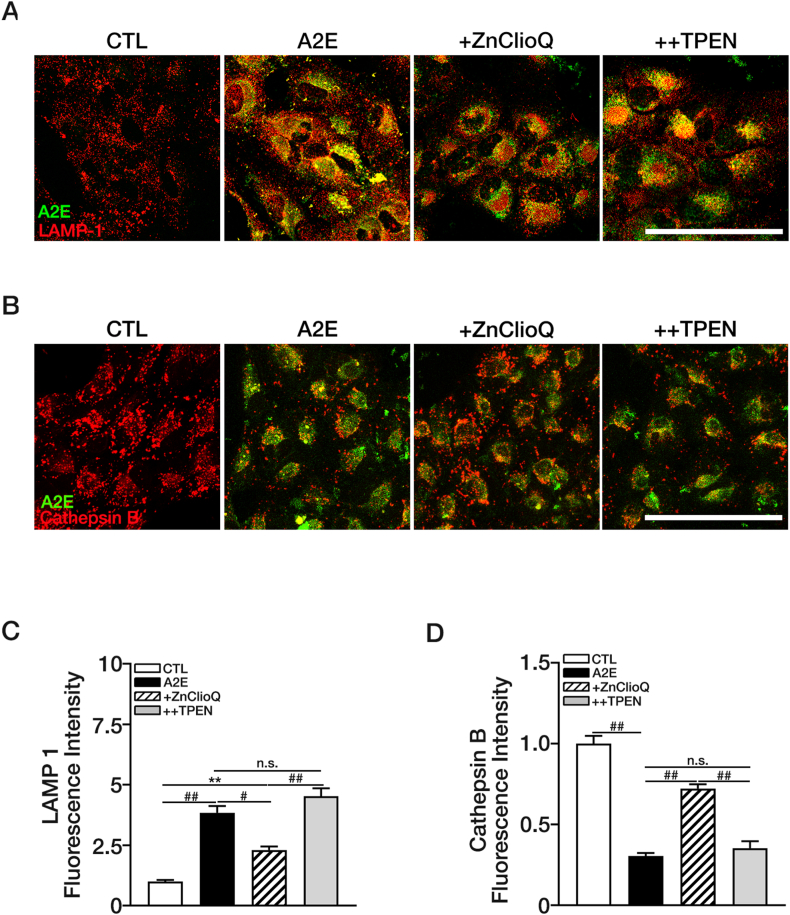
Zinc-induced changes in lysosome enzymatic activity in ARPE-19 cells. (A) Fluorescence images of LAMP-1 immunofluorescence (red) in ARPE-19 cells treated with 100 μM A2E (green dot) and indicated chemicals for 6 h. Original magnification, × 630; scale bar, 100 μm. (B) Confocal live images of Cathepsin B activity (red) in ARPE-19 cells treated with A2E (green dot) and indicated chemicals. Original magnification, × 630; scale bar, 100 μm. (C) Quantification of LAMP-1 fluorescence in (A) using ROI analysis (CTL; n = 5, A2E; n = 4, +ZnClioQ; n = 3, ++TPEN; n = 3, ∗*P* < 0.05, ^#^*P* < 0.005, ^##^*P* < 0.001, one-way ANOVA test). (D) Quantification of Cathepsin B activity in (B) using ROI analysis (CTL; n = 8, A2E; n = 10, +ZnClioQ; n = 10, ++TPEN; n = 9, ^##^*P* < 0.001, one-way ANOVA test). ∗Experimental group = CTL; A2E; + ZnClioQ (A2E + ZnClioQ); ++TPEN (A2E + ZnClioQ + TPEN).

We further investigated whether A2E accumulated in lysosomes. Immunofluorescence staining with the lysosomal marker LAMP-1 showed that most of the A2E accumulated in lysosomes (Fig. 5A). Interestingly, similar to other lysosomal alkalizers such as bafilomycin A1 [59,60] and chloroquine [46,60,61], A2E also markedly increased the number of lysosomes. The ZnClioQ-induced decreases in A2E accumulation in lysosomes are in line with the idea that such an effect is mediated by the reacidification of lysosomes and the subsequent upregulation of lysosomal degradation. As expected, treatment with ZnClioQ reduced the number of lysosomes as well as A2E accumulation, and TPEN effectively abrogated the effects of ZnClioQ (Fig. 5A–C). Moreover, we observed that A2E accumulation was accompanied by reductions in the cathepsin B activity (Fig. 5B–D); ZnClioQ ameliorated the negative effect of A2E on cathepsin B activity, and TPEN reversed the effects of ZnClioQ.

### 3.6 Zinc reduces the accumulation of A2E and its cytotoxicity in RPE cells

**Fig. 6:**
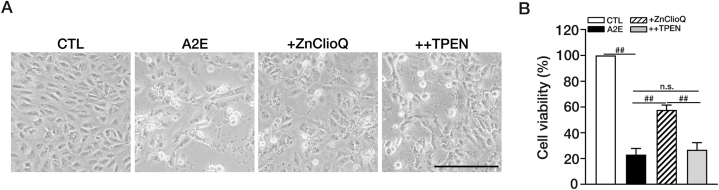
Zinc reduces the accumulation of A2E and its cytotoxicity in RPE cells. (A) Phase-contrast photomicrographs of ARPE-19 cells treated 100 μM A2E, or A2E plus ZnClioQ and or A2E plus ZnClioQ and 0.5 μM TPEN. Original magnification, × 200; scale bar, 200 μm. (B) Cell death measured by MTT assay in ARPE-19 cells. Data are shown as mean ± SEM (CTL; n = 7, A2E; n = 9, +ZnClioQ; n = 7, ++TPEN; n = 9, ^##^*P* < 0.001, one-way ANOVA test). ∗Experimental group = CTL; A2E; +ZnClioQ (A2E + ZnClioQ); ++TPEN (A2E + ZnClioQ + TPEN).

We examined whether ZnClioQ also affected A2E-induced cell death in ARPE-19 cells. Exposure of ARPE-19 cells to 100 μM A2E for 24 h resulted in widespread cell death, as evidenced by detachment and clumping of cells (Fig. 6A). The MTT assay confirmed that A2E exposure for 24 h induced about 80 % of cell death (Fig. 6B). We observed significant attenuation of A2E-induced cell death by the co-addition of ZnClioQ (Fig. 6A and B). Again, TPEN abrogated the protective effect of ZnClioQ, indicating that raising zinc levels was involved in the protective mechanism of ZnClioQ against the A2E cytotoxicity.

### 3.7 ZnClioQ treatment reduced A2E accumulation in the mouse retina

**Fig. 7:**
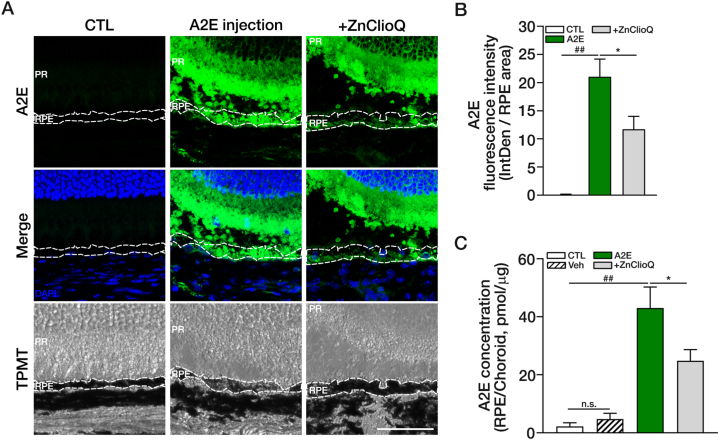
A2E accumulation reduced by ZnClioQ in the mouse retina. (A) Confocal images of A2E in mouse retinal section (A2E; green, DAPI; blue). Original magnification, × 630, × 2 zoom; scale bar, 50 μm. (B) Quantification of A2E fluorescence (A) in RPE layer using ROI analysis. The RPE layer is the black layer with a white line in (A) T-PMT image. (CTL; n = 3, A2E; n = 11, +ZnClioQ; n = 9, ∗*P* < 0.05, ^##^*P* < 0.001, one-way ANOVA test). (C) Quantification of amount of A2E in RPE/choroid tissue using LCMSMS (CTL; n = 8, Veh; n = 8, A2E; n = 9, +ZnClioQ; n = 11, ∗*P* < 0.05, ^##^*P* < 0.001, one-way ANOVA test).

Although ZnClioQ was highly effective in reversing the toxic effects of A2E in ARPE-19 cell culture, likely by normalizing lysosomal pH and function, it remained uncertain whether it would have the same effects in intact retina *in vivo*. To investigate this issue, we injected A2E into the subretinal space in adult mice. After 24 h, we prepared retinal tissue sections. In the A2E-injected eyes, we observed a large amount of A2E accumulation in the subretinal space, the photoreceptor layer, and the RPE layer. Focusing on RPE cells, we found that A2E injection not only increased A2E accumulation but also caused substantial damage to RPE cells (Fig. 7A). However, co-injection of ZnClioQ with A2E significantly reduced both A2E fluorescence accumulation and cell damage in the PRE layer (Fig. 7B). We quantified A2E fluorescence intensity in the RPE layer and confirmed that ZnClioQ reduced A2E accumulation (Fig. 7B). To verify that not only fluorescence but also the actual amount of A2E was altered by ZnClioQ treatment, we measured A2E levels in isolated RPE/choroid tissues using the LCMSMS method. This method revealed that the amount of A2E was approximately 43.03 pmol/μg in the A2E injection group and 24.83 pmol/μg in the ZnClioQ co-injection group (Fig. 7C, Supplementary Fig. S4). Hence, co-injection of ZnClioQ indeed reduced the total amount of A2E accumulated in the RPE layer.

**Fig. 8:**
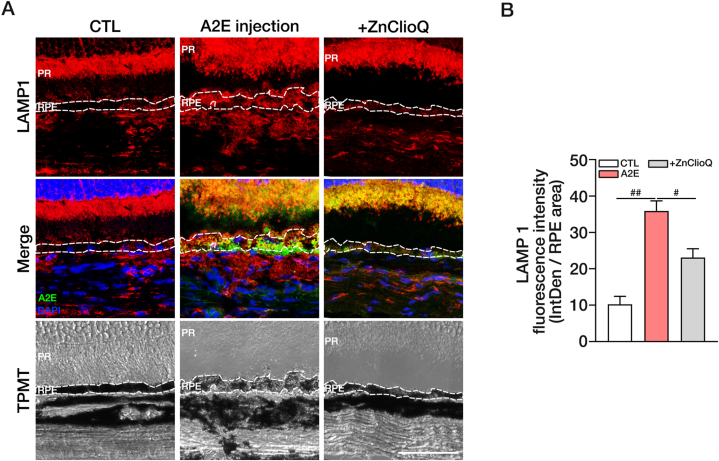
Increased LAMP 1 in A2E injected mice retina and restoration by ZnClioQ. (A) Confocal images of LAMP-1 immunofluorescence (red) in mouse retinal section (A2E; green, DAPI; blue). Original magnification, × 630, × 2 zoom; scale bar, 50 μm. (B) Quantification of LAMP-1 fluorescence (A) in RPE layer using ROI analysis. The RPE layer is the black layer with a white line in (A) T-PMT image. (CTL; n = 5, A2E; n = 10, +ZnClioQ; n = 8, ^#^*P* < 0.005, ^##^*P* < 0.001, one-way ANOVA test).

Finally, we examined whether A2E accumulation would compromise lysosomal markers in this *in vivo* model. As an indirect biomarker, we assessed the level of LAMP-1, which increases when lysosomal functions are compromised [42,46,62,63]. Consistent with lysosomal dysfunction, A2E injection increased the LAMP-1 levels in the RPE layer (Fig. 8). Hence, the protective effect of ZnClioQ against A2E toxicity in RPE cells is not limited to the cell culture system but is extendable to eyes *in vivo*.

## 4 Discussion

The complicated, multifactorial disease known as AMD is typified by retinal pigment epithelial cells and photoreceptor degeneration [1,2,4]. AMD’s etiology and pathophysiology involve chronic inflammation, decreased choroidal blood flow, chronic oxidative stress, and hereditary factors [4,64,65]. Specifically, insoluble aggregates accumulate in extracellular space in dry AMD, and the loss of RPE cells' degradative function leads to an additional accumulation of lipoprotein aggregates [7,9,10]. One of the major lipofuscins in RPE cells is A2E [[17], [18], [19]], which is derived from photoreceptor outer segments and accumulates within the lysosomes of RPE cells and incurs harmful effects on the retina [14,17,18,66]. Lysosomes are the primary mediators of lipoprotein aggregates degradation [14,24,32,52]. Importantly, the primary degradative enzymes of RPE cells are highly dependent on pH, and lysosomal enzymes are only capable of functioning in a narrow range of acidic pH.

Zinc, one of the most abundant metals in the eye, plays a crucial role in both physiological and pathological processes [38,40,67]. Plasma zinc levels decrease with age, contributing to the pathophysiology of various conditions, including neurodegenerative diseases and age-related macular degeneration [[68], [69], [70]]. Notably, numerous studies have implicated lysosomal dysfunction as a common underlying pathogenic change in these age-related diseases [[71], [72], [73]]. Decreased lysosomal activity results in the accumulation of toxic proteins and lipids. In age-related macular degeneration, the accumulation of compounds such as lipofuscin and A2E increases lysosomal pH, further impairing lysosomal degradation and leading to additional waste accumulation. When considering the above studies and those highlighting the role of zinc in regulating lysosomal pH and function, it seems plausible that age-dependent decreases in intracellular zinc levels may contribute to age-related lysosomal dysfunction. Conversely, increasing intracellular and lysosomal zinc levels activates transcription factor EB (TFEB) and promotes the assembly of V-ATPase, a critical enzyme for lysosomal acidification and function [41]. Several recent studies provide compelling evidence that zinc can significantly enhance lysosomal function [42,74].

In the present study, we established an *in vitro* model for AMD by focusing on the lysosomal dysfunction caused by A2E. Previous studies have shown that engulfment of A2E by RPE cells may underlie the lysosomal dysfunction that leads to the formation of drusen and the development of AMD [13,75]. Indeed, we observed that exposure of ARPE-19 cells to A2E mimicked some of the AMD pathologies such as pH alteration and dysfunction of lysosomes. Upon exposure, ARPE-19 cells gradually took up A2E over an hour (Fig. 1). Concurrently, lysosomal pH gradually shifted to the alkaline direction (Fig. 2). These results are consistent with previous reports showing that A2E may increase lysosomal pH by inhibiting vATPase, which is the primary proton pump for lysosomes [25,[76], [77], [78]]. As in the case with other vATPase inhibitors such as bafilomycin A1 [59,60,78], A2E increased not only pH but also the number of lysosomes as reflected by an increase in the level of LAMP1 (Fig. 5A–C), which may be a compensatory response of the RPE cells. However, likely due to the markedly diminished function as exemplified by a decrease in cathepsin B activity (Fig. 5B–D), simply increasing the number of lysosomes may not be sufficient in preventing the accumulation of A2E.

However, treatment with ZnClioQ to raise free zinc levels in the cytosol and lysosomes had a notable protective effect against the toxic effects of A2E including lysosomal pH changes, lysosomal enzyme dysfunction (cathepsin B activity), and cell death. It is yet unclear how lysosomal zinc is able to counteract the effects of A2E. In other cell types such as cerebral astrocytes, ZnClioQ showed similar effects on lysosomes [42,58,79]. Therefore, regardless of the mechanism, increasing lysosomal zinc may be an effective measure in nullifying A2E toxicity in RPE cells, which may be specifically relevant in AMD pathogenesis. Likely due to an increase in lysosomal degradative function, zinc clioquinol markedly reduced A2E levels in ARPE-19 cells. Although a decrease in intracellular A2E levels can occur if zinc clioquinol reduces A2E uptake during co-exposure, our washout experiment, in which zinc clioquinol was added after thorough washout, showed that zinc clioquinol was still effective in reducing A2E accumulation. To further verify this, we measured A2E levels in cells by mass spectroscopy and obtained the same results. Hence, it is likely that zinc clioquinol increased the degradation of A2E by enhancing lysosomal functions.

Since there is no reliable *in vivo* model for AMD that includes A2E toxicity to retinal cells, it is challenging to directly assess the impact of zinc-clioquinol on A2E levels *in vivo*. Hence, we attempted to design an *in vivo* experiment by injecting A2E into the subretinal space of mice. Even in this relatively simple model of A2E accumulation, the co-injection of zinc-clioquinol significantly decreased the amount of A2E that accumulated in RPE cells. It is ideal to measure lysosomal pH in retinal tissue, but we found that lysosomal fluorescent dyes do not work well in retinal sections. Instead, we examined the level of LAMP1, which increases with changes in lysosomal pH. Here, A2E treatment markedly increased LAMP1 immunofluorescence. Again, co-treatment with zinc clioquinol substantially reduced the effect of A2E on LAMP1 levels. Hence, increasing zinc levels in lysosomes of RPE cells may help decrease A2E levels in AMD, likely by improving lysosomal degradation.

**Fig. 9:**
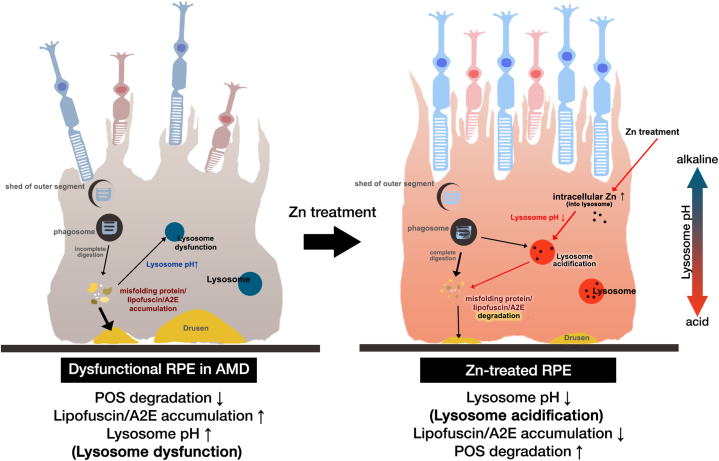
Proposed mechanism of the effects of intracellular zinc on lysosomal alkalinization/dysfunction and AMD due to retinal lipofuscin in RPE. Diagram of zinc-induced lysosomal activation in RPE. In AMD, the RPE shows a diminished capacity to degrade photoreceptor outer segments (POS), leading to an increase in lysosomal pH and an accumulation of lipofuscin. Increasing intracellular zinc levels restored both the acidity and the degradative function of lysosomes in the RPE. Regulation of intracellular zinc may be a viable target for the treatment of AMD.

The current study demonstrated that increasing zinc levels, particularly in lysosomes, would help mitigate the cytotoxic effects of AMD-associated agents such as A2E or lysosomal alkalizers (Fig. 9). Some studies have suggested that aberrant protein aggregates accumulating to a pathological degree is a common feature of AMD and neurodegenerative diseases, including Parkinson’s disease [[80], [81], [82], [83]], Alzheimer’s disease [[84], [85], [86], [87]], Huntington’s disease [83,87], and amyotrophic lateral sclerosis. As demonstrated in the present study, as well as in some *in vitro* models of neurodegenerative diseases, zinc treatment enhances the functional activity of lysosomes [42,88,89]. Considering that dry AMD and neurodegenerative diseases have similar pathogenic processes, including lysosomal dysfunction, therapeutic strategies that increase lysosomal zinc levels may be effective in restoring lysosomal function in these conditions.

## CRediT authorship contribution statement

**Jeong A. Choi:** Writing – original draft, Writing – review & editing, Visualization, Validation, Project administration, Methodology, Investigation, Formal analysis, Conceptualization. **Bo-Ra Seo:** Validation, Investigation. **Jae-Young Koh:** Writing – review & editing, Supervision, Funding acquisition, Conceptualization. **Young Hee Yoon:** Writing – review & editing, Supervision, Funding acquisition, Conceptualization.

## Ethics statement

The animal experiment protocol was approved by the Institutional Animal Care and Use Committee (IACUC) of Asan Institute for Life Sciences, University of Ulsan College of Medicine, Seoul, Korea (approval number: 2021-12-280).

## Data availability statement

The datasets generated during and/or analyzed during the current study are available from the corresponding author upon reasonable request.

## Funding

This research was supported by the Basic Science Research Program through the 10.13039/501100003725National Research Foundation of Korea (NRF) funded by the 10.13039/501100002701Ministry of Education (2017R1D1A1B05028221) and the Korea 10.13039/100018696Health Technology R&D Project through the 10.13039/501100003710Korea Health Industry Development Institute (10.13039/501100003710KHIDI) and 10.13039/100020206Korea Dementia Research Center (KDRC), funded by the 10.13039/100009647Ministry of Health & Welfare and 10.13039/501100014188Ministry of Science and ICT (HI20C0206).

## Declaration of competing interest

All authors declare that they have no known competing financial interests or personal relationships that could have appeared to influence the work reported in this paper.
