# Supplementary material for: Protective effect of zinc against A2E-induced toxicity in ARPE-19 cells: Possible involvement of lysosomal acidification
Source: Heliyon. 2024 Oct 11;10(21):e39100. doi: 10.1016/j.heliyon.2024.e39100 (PMC11550603; doi:10.1016/j.heliyon.2024.e39100)
Supplement: Multimedia component 1 [file mmc1.docx]

**Supplementary Data**

**Supplementary Figure S1**


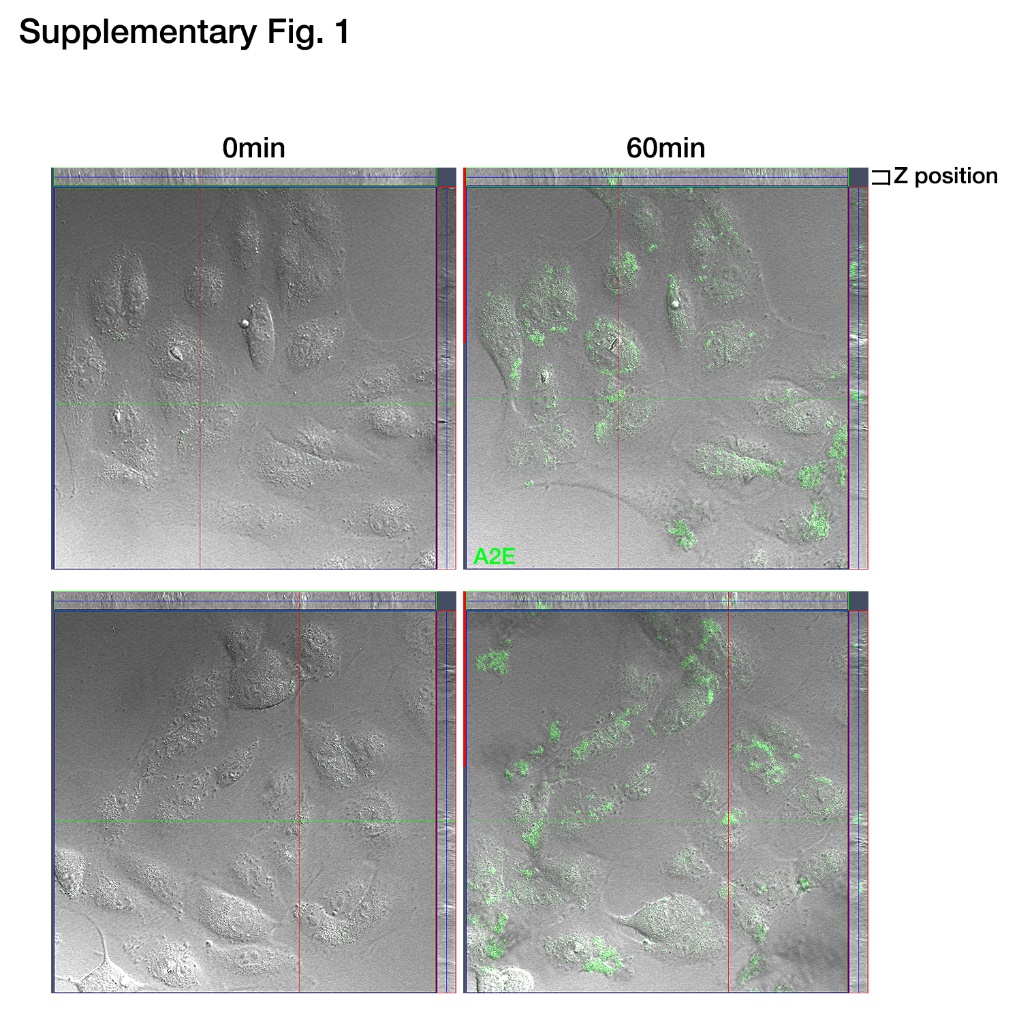


**Fig S1. Intracellular accumulation of A2E**

Confocal live images showing the accumulation of A2E (green) in intracellular area following treatment with 100 μM A2E. Original magnification, × 400; Z-stack; 10 μm

**Supplementary Figure S2**


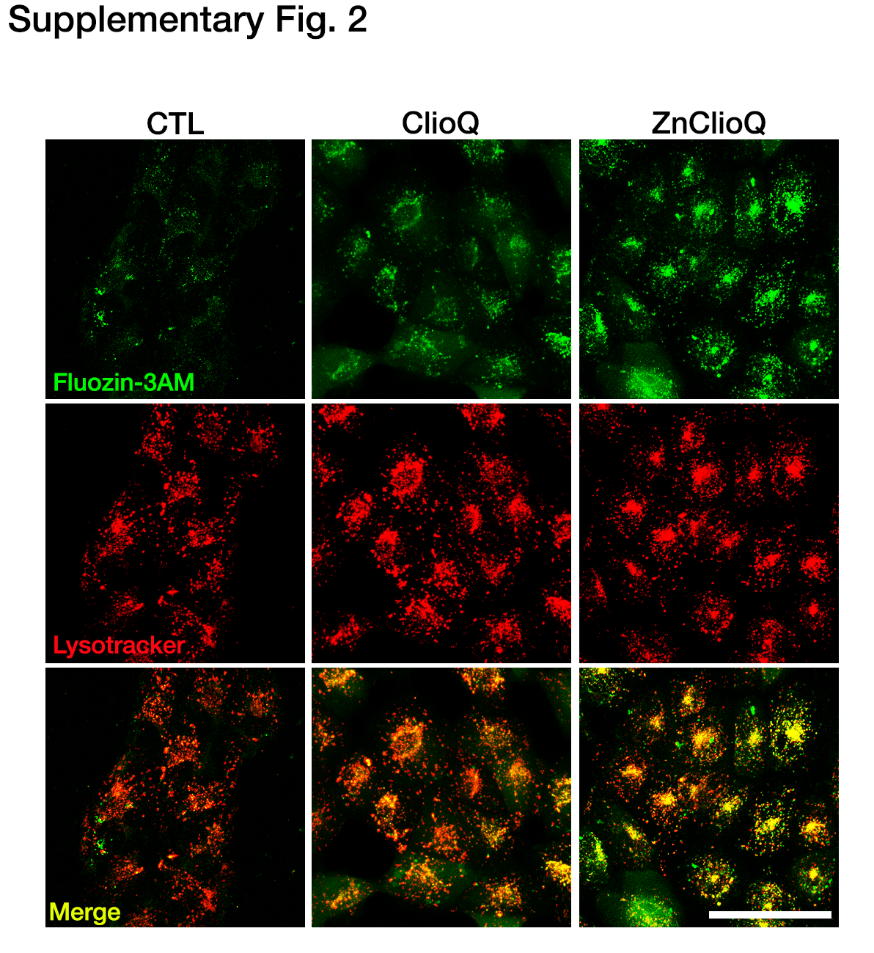


**Fig S2. Clioquinol is a potent zinc ionophore in ARPE-19 cells.**

Confocal images of ARPE-19 cells stained with Fluozin-3 AM (Zn^2+^ selective marker) and Lysotracker. The cells were treated with 1 μM ClioQ or ClioQ plus 0.5 μM zinc (ZnClioQ) for 1 h in normal MEM. Original magnification, × 630; scale bar, 100 μm.

**Supplementary Figure S3**


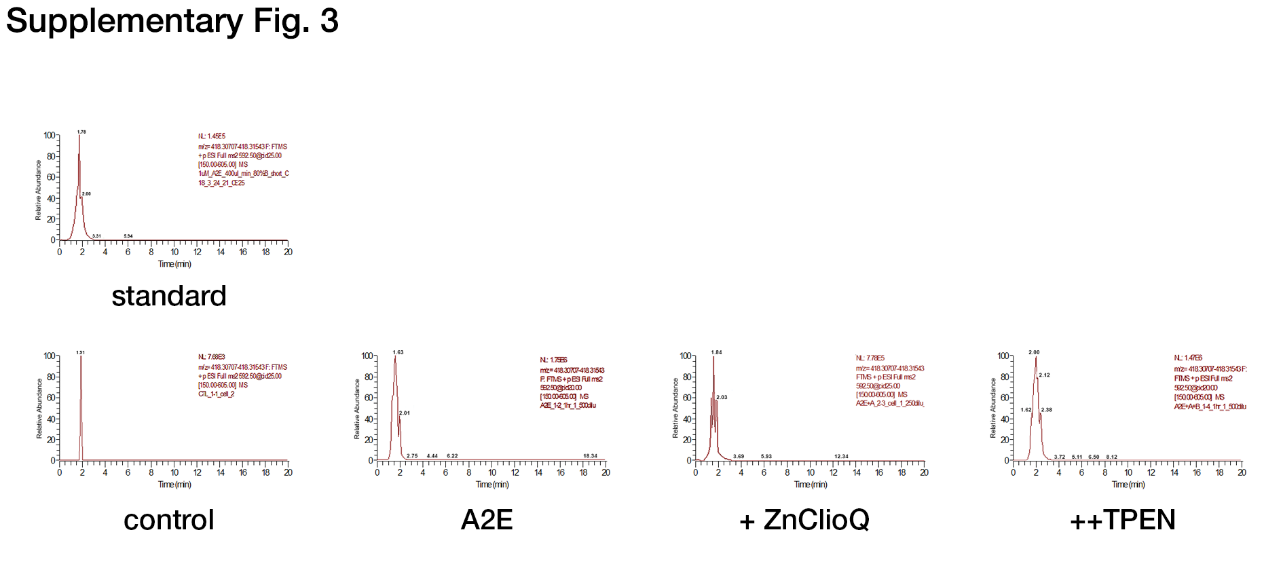


**Fig S3. Intracellular A2E measurement using LCMSMC**

The extracted chromatogram from the mass spectrometry analysis of A2E (m/z 592). The extracts were from non-treated, A2E alone, A2E plus ZnClioQ, or A2E plus ZnClioQ and TPEN treated APRE-19 cells. The A2E peak is indicated with an asterisk. (upper; total mass, lower; A2E peak)

**Supplementary Figure S4**


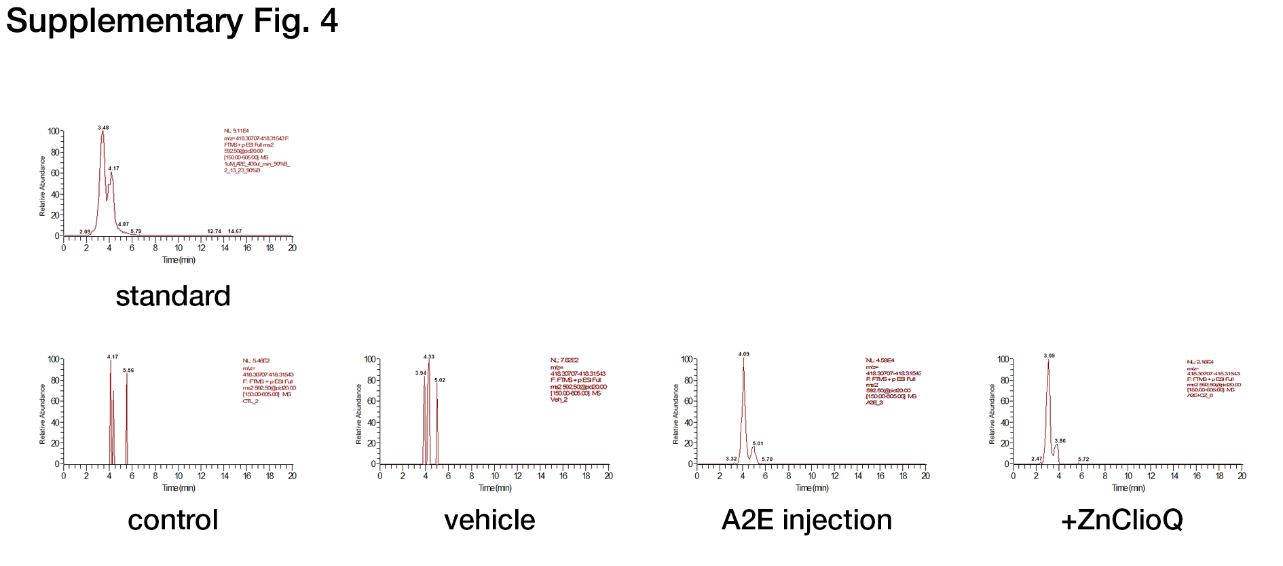


**Fig S4. A2E measurement using LCMSMC in mouse RPE/Choroid tissue**

The extracted chromatogram from the mass spectrometry analysis of A2E (m/z 592). The extracts were from control, vehicle, A2E alone or A2E plus ZnClioQ injected mouse RPE/choroid. The A2E peak is indicated with an asterisk. (upper; total mass, lower; A2E peak)
